# Global burden of SARS-CoV-2 infection, hospitalization and case fatality rate among COVID-19 vaccinated individuals and its associated factors: A systematic review and meta-analysis protocol

**DOI:** 10.1371/journal.pone.0272839

**Published:** 2022-08-09

**Authors:** Alex Durand Nka, Aude Christelle Ka’e, Yagai Bouba, Ezechiel Ngoufack Jagni Semengue, Michel Carlos Tommo Tchouaket, Désiré Takou, Willy Pabo, Nadine Fainguem, Samuel Martin Sosso, Vittorio Colizzi, Carlo-Federico Perno, Joseph Fokam

**Affiliations:** 1 Chantal BIYA International Reference Centre for Research on HIV/AIDS Prevention and Management, (CIRCB), Yaoundé, Cameroon; 2 University of Rome “Tor Vergata”, Rome, Italy; 3 Evangelical University of Cameroon, Bandjoun, Cameroon; 4 National AIDS Control Committee (NACC), Yaoundé, Cameroon; 5 Faculty of Sciences, University of Buea, Buea, Cameroon; 6 Children Hospital Bambino Gesu’ IRCCS, Rome, Italy; 7 Faculty of Health Sciences, University of Buea, Buea, Cameroon; Keele University, UNITED KINGDOM

## Abstract

**Background:**

COVID-19 has been the most important public health concern worldwide since 2020. Several vaccines are now available to help in controlling COVID-19 associated morbidity and mortality. This study will aim to provide the global and regional prevalence of SARS-CoV-2 infection as well as an estimate of disease severity among COVID-19 vaccinated individuals.

**Materials and methods:**

In order to determine the global burden of SARS-CoV-2 infection among vaccinated individuals, we will systematically extract and review papers from PubMed/MEDLINE, Excerpta Medica database (EMBASE), Cochrane Central Register of Controlled Trials (CENTRAL), Science direct and Cumulative Index to Nursing and Allied Health Literature (CINAHL). All the studies describing the prevalence and/or disease severity (hospitalization and case fatality rate) data of COVID-19 among individuals who received a partial or complete dose of WHO-approved COVID-19 vaccines will be eligible. A random effect model will be used to calculate the pooled prevalence and to estimate the disease severity. Subgroup analysis will be performed to explore the association between the number of vaccine doses received and the COVID-19 burdens.

**Discussion:**

This systematic review and meta-analysis will provide the global estimate data on pooled prevalence, hospitalization and case fatality rates of COVID-19 among vaccinated individuals. Moreover, the factors associated with reinfection and disease severity will be equally investigated in the meta-analysis. The results of this study will contribute in the understanding and estimation of the global burden of COVID-19 among vaccinated individuals. Findings will provide meaningful information for the success of the current global rollout of COVID-19 vaccination strategies and pave the way for future interventions.

**Systematic review registration:**

CRD42021273074.

## Background

The novel coronavirus, SARS-CoV-2 is responsible for the coronavirus disease 2019 (COVID-19) and was first reported on December 31, 2019 in Wuhan, China [1, 2]. The COVID-19 infection spread rapidly worldwide, and the WHO then declared it as a pandemic [3]. Transmitted person-to-person, the genetic variants of SARS-CoV-2 have emerged and circulating worldwide, by causing important morbidity and mortality [4]. By September 27, 2021, WHO reported about 231 million COVID-19 cases and more than 4.7 million related deaths [5].

The emergency use approval of the first vaccines by WHO and FDA in 2020 brought a lot of hope in the control and elimination of the COVID-19 pandemic. So far, several COVID-19 vaccines based either on live adenovirus (Oxford-Astrazeneca, Covishield and Johnson & Johnson), inactivated (Sinopharm) or ribonucleic acid (Pfizer, Moderna) have been developed [6]. As of today, six COVID-19 vaccines have been approved by WHO and are used worldwide [6]. Of them, five require a booster dose (Pfizer, Moderna, Sinopharm, Oxford-Atrazeneca and Covishield) and one (Johnson & Johnson) require only one shot [7]. Even though these COVID-19 vaccines are safe even among those with pre-existing comorbidities [8], their efficacy varies only from 52% to 89% at preventing infection from both ancestral and Alpha variants; and from 73% to 94% at protecting against the severe form of disease [9–13]. The efficacy of these vaccines to prevent from COVID-19 infection for the Beta, and Gamma and Delta ranges from 60 to 94% and 49 to 80%, respectively [9]. So far, globally, as from September 27, 2021, close to 6 billion vaccine doses were administered; about 3.3 billion individuals received at least one dose and 2.4 billion persons are fully vaccinated [5]. In order to ensure vaccine equity, 92 low and middle-income countries were able to access COVID-19 vaccine through the COVID-19 vaccines global access initiative (COVAX) [14].

Another important aspect is the durability of the immunity against COVID-19 infection. Some data suggest that it takes weeks for the body to mount effective immunity level after receiving COVID-19 vaccine [9]. Similarly, according to the US CDC, full protection occurs two weeks after the second dose of the Pfizer or Moderna COVID-19 vaccines, or two weeks after the single-dose Johnson & Johnson vaccine [13]. On the other hand, the period for how long the vaccinated individuals remain protected is still to be fully understood [15], posing therefore the problem of COVID-19 infection among these individuals.

In this regard, as the vaccination rollout was ongoing, some studies reported the cases of SARS-CoV-2 infection among vaccinated health care personnel [16], including COVID-19 vaccine breakthrough infections associated with large public cluster [17–19]. Another study conducted amidst the pandemic dominated by the circulation of delta variant in the United Kingdom showed a high hospitalization and death rates among vaccinated individuals [20]. The study reporting on the burden of COVID-19 among vaccinated individuals are scare and fragmented. To the best of our knowledge, there is no summary of the global prevalence, case fatality rate and hospitalization rate among vaccinated people. The global and regional data on the burden of COVID-19 among vaccinated people is now crucial to adjust for the vaccination strategies, especially in the present context which is marked by the emergence of new SARS-CoV-2 variants that might be able to escape the immunity acquired through vaccination or following an infection by the SARS-CoV-2 virus. Thus, this systematic review and meta-analysis will seek to investigate on the global prevalence, case fatality and hospitalization (severe COVID-19 outcomes) rates of COVID-19 among vaccinated individuals and the factor associated with these parameters.

## Materials and methods

### Design and registration

This systematic review and meta-analysis protocol followed the recommendation of the Preferred Reporting Items for Systematic Reviews and Meta-Analysis (PRISMA) [21] and PRISMA-P (https://prisma-statement.org/Extensions/Protocols) guidelines. This review protocol was registered in the Prospective Register of systematic Reviews (PROSPERO) and is available on: https://www.crd.york.ac.uk/prospero/display_record.php?ID=CRD42021273074. For the complete PRISMA checklist, see the S1 File.

### Inclusion criteria

The papers that will considered in our analysis should include:

1. **Type of studies:** Randomized and non-randomized trials, cohort and cross-sectional studies evaluating the prevalence and COVID-19 disease severity among vaccinated individuals.
2. **Type of participants:** We will consider studies conducted among COVID-19 vaccinated individuals according to age (adolescents, adults), gender, profession, type of vaccine, number of doses administered and geographical location (countries). The participants will be classified into the following sub-groups: fully vaccinated, partially vaccinated, peoples with booster doses, people with breakthrough infections and vaccinated.
3. **Intervention:** COVID-19 vaccination will be our intervention of interest. Studies focusing on vaccinated people by Oxford-AstraZeneca, Moderna, Pfizer, Johnson and Johnson, Covishield, Covaxin and Sinopharm as approved by the World Health Organization (WHO). [6]
4. **Comparators:** Type of vaccines, number of doses (fully or partially vaccinated, booster doses, breakthrough infections before vaccination), sociodemographic parameters if available:
  Gender (Male/Female), age group (12 to 19 years, 19 to 39 years, 40 to 64 years and ≥65 years), race/ethnicity, profession (health care personnel *versus* others), presence of comorbidities (immunosuppression, chronic pulmonary disease, chronic leaver disease, chronic kidney disease, chronic neurologic disease, diabetes, chronic cardiac disease, overweight/obesity and others), countries and the infecting variant as classify by CDC [22] when available.
5. **Types of outcomes:** Primary outcome will be the COVID-19 infection and the secondary outcomes will be the hospitalization (severe COVID-19 outcome) and death. Severe COVID-19 outcomes will be defined as hospitalization with diagnosis of acute respiratory failure, need for noninvasive ventilation (NIV), admission to an intensive unit including persons requiring invasive mechanical ventilation.
6. **Report characteristics:** We will include studies that have been published in English or French from January 2020 till the date the data will be queried from various sources.

#### Exclusion criteria

Case reports, letters, comments, reviews, systematic reviews and meta-analysis, and editorials studies will be excluded.

### Search strategy

A systematic literature search will be performed in PubMed/MEDLINE, Excerpta Medica database (EMBASE), Cochrane Central Register of Controlled Trials (CENTRAL), Science direct and Cumulative Index to Nursing and Allied Health Literature (CINAHL) using the key terms: "COVID-19", coronavirus, "corona virus", “coronaviruses”, "2019-nCoV", "SARS-CoV", “Severe Acute Respiratory Syndrome”, “SARS-CoV-2”, “novel coronavirus”, “prevalence”, “positivity rate”, “Hospitalized individuals”, “Hospitalized people”, “Hospitalization”, “Immunization”, “vaccinated individuals”, “vaccinated people”, “vaccinated person”, “case fatality rate”, “death”, “severity” and “hospitalization” linked by the Boolean operators “OR” and “AND” (S2 File shows the detailed search strategy for Pubmed and Embase). Additional related articles will be retrieved manually from the reference lists of included studies and Google Scholar and will be then critically evaluated for inclusion.

### Selection of studies for inclusion in the review

Articles retrieved from databases will be independently selected by two authors (AND, ACK) using “SysRev” (https://sysrev.com/), a software for a systematic review production tool for title/abstract screening. Any disagreement will be solved by discussion, consensus, or will involve a third review author (JF or YB) as an arbitrator. Two review authors (AND, ACK) will independently evaluate the full text of the selected records. Discrepancies will be resolved by consensus or by an arbitration of a third review author (JF or YB). The agreement between the two first review authors will be estimated by Cohen’s kappa coefficient.

### Data extraction and management

After the screening of published articles for eligibility, a google form questionnaire will be used for the extraction of relevant data and information. The following items will be obtained from each study (if described): primary author, year of publication, country, study design and period, number of persons screened for the COVID-19, number of COVID-19 positive individuals, the type of variant identified among COVD-19 positive person, the type of vaccine, the number of doses received, data on hospitalization, the number of deaths, data on comorbidity if available, and data relating to age, gender and profession.

All records from the various sources included in our search strategy will be combined, uploaded into the reference management software Zotero® (version 5.0.85). Duplicates will be removed from the analysis.

### Data synthesis

Data analysis will be performed using the “meta” and “metafor” packages of the R statistical software through the RStudio interface (V.3.4.4, R Foundation for Statistical Computing, Vienna, Austria) [23, 24]. Heterogeneity of the studies will be estimated using H statistic test and will be quantify by I² [25]. The I^2^ value will calculate the percentage of total variation across studies due to true between-study differences rather than chance. The degree of heterogeneity with values of 0%, 18%, 45%, and 75% with p < 0.05 will designate a none, low, moderate and high heterogeneity respectively [26]. The pooled prevalence and case fatality rate at 95% confidence intervals (95% CI) will be calculated using the “meta-prop command” by random effect model [27]. Subgroup analyses according to the study design, geographical area as defined by the United Nations [28], profession, gender, type of vaccine, comorbidities, and number of doses administered will be employed to adjust the variations in the pooled estimate of the prevalence and hospitalization rate and case fatality rate. Results will be considered as statistically significant if p <0.05. The GRADE approach will be used to rate the certainty of evidences as “high”, “moderate”, “low” and “very low” (S3 File).

### Quality assessment and risk of bias

The evaluation of included studies for the risk of bias will be done using ROBINS-1 [29, 30], a tool for assessing the risk of bias in non-randomized studies for interventions. ROBIS [RoB 2.0] [29, 30] will be used for randomized controlled trial studies. For observational studies, we will used NIH quality assessment tool for observational cohort and cross-sectional studies (NOS) [31]. Discrepancy in the risk of bias assessment among the review authors will be solved by discussion and consensus, or by arbitration of a third review author.

The publication bias will also be assessed by visual inspection of the asymmetry of the funnel plot and the Egger test with the value of p <0.1 indicating a potential bias [32].

## Discussion

Vaccination is key for the effective control of the COVID-19 pandemic. The durability of the immunity acquired through COVID-19 and the risk of infection among vaccinated individuals represent important threats to the successful achievement of this crucial prevention strategies, as it is the case with other infectious diseases. Our analysis will aim at providing the burden of COVID-19 among vaccinated individuals. Moreover, we will aim at identifying the factors associated with the acquisition of the infection and the risk of experiencing disease severity among this group of people. We are confident that our results will be useful to the scientific community and policy makers in the global estimation of the real burden of COVID-19 among vaccinated individuals. The sub analysis that will be performed will better help in understanding if there are regional variation or if it is related to the types of vaccines received or the number of doses received or if there are other individuals’ characteristics on which interventions might be made in order to mitigate this problem. We are aware of the potential limitations of this review that might be related to factors such as study heterogeneity; but appropriate statistical models will be used to control for such problem during meta- analysis. An important problem that might be encountered is the incompleteness of data in the selected studies. Parts of the problem will be solved by reaching out to study corresponding author, with no guaranty of retrieving all the data if the authors did not collect the data. Important protocol adjustments will be documented, taken into consideration while analyzing the data, and discussed consequently in the final manuscript. Our findings will be presented at relevant scientific conferences and published in a peer-review journal to ensure the quality of the results and for wider diffusion of the results to inform policy and all those who might be interested.

## Supporting information

S1 File PRISMA-P 2015 checklist. (DOCX)

S2 File Search strategy. (DOCX)

S3 File Assessing the quality of evidences and the strength of recommendations. (DOCX)
