# Supplementary material for: Global burden of SARS-CoV-2 infection, hospitalization and case fatality rate among COVID-19 vaccinated individuals and its associated factors: A systematic review and meta-analysis protocol
Source: PLoS One. 2022 Aug 9;17(8):e0272839. doi: 10.1371/journal.pone.0272839 (PMC9362926; doi:10.1371/journal.pone.0272839)
Supplement: S2 File — (DOCX) [file pone.0272839.s002.docx]

Additional file 2: Search strategy in Medline (Pubmed) and Embase

| **Database**  **PubMed** | **Search** | **Query** |
| --- | --- | --- |
|  | #1 | COVID-19 OR SARS-CoV-2 OR "COVID-19" OR "coronavirus, "corona virus" OR "coronaviruses" OR «2019-nCoV" OR "SARS-CoV" OR" Severe Acute Respiratory Syndrom" OR "SARS-CoV-2" OR "novel coronavirus" |
|  | #2 | “Positivity Rate” OR “Case Fatality Rate” OR “Death” OR “prevalence” OR “Hospitalized individuals” OR “Hospitalized people” OR “Hospitalization” OR OR “case fatality rate” OR “death”, OR “severity” OR “hospitalization” |
|  | #3 | “Vaccinated People” OR “Vaccinated individuals” OR Vaccine OR “Vaccination” OR “vaccinated individuals” OR “vaccinated people” OR “vaccinated person” |
|  | #4 | #1 AND #2 AND #3 |
| **Embase**  **(Ovid interface)** |  | exp COVID-19 |
|  |  | exp SARS-CoV-2 |
|  |  | exp “SARS-CoV-2 infection” |
|  |  | exp “Novel Coronavirus” |
|  |  | Or/1-4 |
|  |  | exp Prevalence |
|  |  | exp “Positivity rate” |
|  |  | exp “case fatality rate” |
|  |  | exp “Hospitalized individuals” |
|  |  | exp Death |
|  |  | Or/6-9 |
|  |  | exp “Vaccinated people” |
|  |  | exp “Vaccinated individuals” |
|  |  | exp Vaccine |
|  |  | exp Vaccination |
|  |  | Or/10-13 |
|  |  | 5 and 10 |
|  |  | 10 and 15 |
