# Supplementary material for: Global burden of SARS-CoV-2 infection, hospitalization and case fatality rate among COVID-19 vaccinated individuals and its associated factors: A systematic review and meta-analysis protocol
Source: PLoS One. 2022 Aug 9;17(8):e0272839. doi: 10.1371/journal.pone.0272839 (PMC9362926; doi:10.1371/journal.pone.0272839)
Supplement: S3 File — (DOCX) [file pone.0272839.s003.DOCX]

**Additional file 3**: Assessing the quality of evidences and the strength of recommendations.

| **Types of studies** | **Risks of bias** | **Interpretation** | **Quality of evidence** | **Strength of the recommendation** |
| --- | --- | --- | --- | --- |
| Randomized studies | Low risk of bias | Most information is from studies at low risk of bias. | High | Strong |
|  | Unclear risk of bias | Most information is from studies at low or unclear risk of bias. | Moderate | Moderate |
|  |  |  | Low |  |
| Non-randomized studies | High risk of bias | The proportion of information from studies at high risk of bias is sufficient to affect the interpretation of results. | Very Low | Weak |
